# A moderated-mediation analysis of abusive supervision, fear of negative evaluation and psychological distress among Egyptian hotel employees

**DOI:** 10.1007/s12144-022-03822-4

**Published:** 2022-10-26

**Authors:** Kareem M. Selem, Erhan Boğan, Ali Elsayed Shehata, Hanan Ahmed Mohamed

**Affiliations:** 1 Hotel Management Department, Faculty of Tourism and Hotels, Suez Canal University, Ismailia, 41522 Egypt; 2 Tourism Guiding Department, Faculty of Tourism, Adıyaman University, Adıyaman, Turkey; 3 Marketing Department, Faculty of Business Administration, Shaqra University, Shaqra, Saudi Arabia; 4 Department of Psychology, Faculty of Arts, Assiut University, Assiut, Egypt

**Keywords:** Abusive supervision, Cognitive appraisal theory, Hotel employees, Psychological distress, Negative reciprocity

## Abstract

By integrating cognitive appraisal and social exchange theories, this paper examines the linkage of supervisors’ abusive reactions with psychological distress through their subordinates’ fear of negative evaluation. This paper also investigates the moderating role of negative reciprocity. Data were gathered from 412 employees at 15 five-star hotels in Egypt and analyzed using PROCESS 4.0 macro in SPSS (Model 5). The findings revealed that abusive supervision was strongly positively connected with psychological distress caused by FNE. Furthermore, negative reciprocity may reduce the abusive supervision-psychological distress relationship. The positive effect of abusive supervision on psychological distress was weaker among hotel employees who held high levels of negative reciprocity. The PROCESS and narratological results highlight the need of taking negative reciprocity into account while investigating undesirable workplace behavior (i.e., abusive acts of supervisors) in the hospitality context by providing fruitful theoretical and practical contributions.

**Supplementary Information:**

The online version contains supplementary material available at 10.1007/s12144-022-03822-4.

## Introduction

Nowadays, organizations pay much more care and attention to supervision due to the importance of leadership in the organizational climate. Adopting Khan’s definition (2015, p. 64), supervision is defined as an expert, technical service that is specifically designed to increase the productivity of the many groups of employees it oversees, with a focus on time management and preparation. Instead, Raza et al. (2019) show that supervision is a process for businesses using their strategic resources to accomplish their predetermined long-term and short-term goals and objectives. Due to the prevalence of unethical leadership behaviors in the organizational environment, researchers are interested in investigating abusive supervision in detail (Zhang & Bednall, 2016), which is described as “*subordinates’ perceptions of the extent to which supervisors engage in the sustained display of hostile verbal and nonverbal behaviors*” (Tepper, 2000, p.178).

Abusive supervision has a negative effect on employee performance as well as that of the entire organization (Ampofo et al., 2022). Generally speaking, it is intimately connected to a variety of negative psychological outcomes, such as powerlessness and decreased self-efficacy (Rasheed et al., 2021). Some key attributes of the hospitality and tourism industries require a detailed examination of the concept of abusive supervision. These attributes include the organizational structures (centralized and hierarchical), the large number of temporary employees, the need to employ a large number of employees with different cultures, and high interaction with customers (Yu et al., 2020). According to Lyu et al. (2016a), abuse of supervision was negatively correlated with customer-focused citizenship behavior trough work engagement at the Chinese hotels.

Hon and Lu (2016) revealed that abusive supervision positively affected subordinates’ abusive behavior and that subordinates’ abusive behavior negatively affected service performance. Wang et al. (2020) indicated that Employee silence mediated the influence of abusive supervision on work engagement. Mackey et al. (2015) asserted that the linkage of abusive supervision with coworker-directed deviance was more pronounced among highly empowered hospitality employees. Despite the attempts to interpret the linkage of abusive supervision with employee responses in the hospitality industry, there are still some research gaps that need to be investigated. For instance, Yu et al. (2020) and Lyu et al. (2016a, b) called for research to explore the underlying mechanisms, processes, and moderators in the abusive supervision-employee responses link.

The current study addresses these calls by investigating the mediating effect of fear of negative evaluation (FNE) and the moderating effect of negative reciprocity in the abusive supervision-psychological distress linkage. FNE refers to “*apprehension about others’ evaluations, distress over their negative evaluations; avoidance of evaluative situations; and the expectation that others would evaluate one negatively*” (Watson & Friend, 1969, p. 449). Previous research has found that people with high FNE are more distressed than people with low FNE (Shafique et al., 2017). According to the current study, employees who are subjected to abusive supervision are hesitant to engage in social interactions where they may feel they are being judged negatively by others, which may increase their psychological distress. Nonterah et al. (2015) supported the mediating role of FNE in the linkage of academic stress with anxiety and depression. FNE results in a fear of social appreciation, which in turn may lead to psychological distress among the employees (Shafique et al., 2017).

Additionally, given the role of negative reciprocity as a moderator variable, individuals respond in the same way they are treated based on SCT theory (Blau, 1964). In other words, people frequently reciprocate unfair treatment (Cropanzano & Mitchell, 2005). Negative reciprocity entails a tendency where something given influences or obligates the other party to return an equivalent gesture (Chhabra, 2020). According to Mitchell and Ambrose (2007), the negative reciprocity plays a key role in identifying the indirect effect of abusive supervision on psychological distress through FNE. Positive reciprocity beliefs are prioritized in the majority of studies (Boğan & Dedeoğlu, 2022; Cheng et al., 2022), which foster stable relationships through being attentive, recognizing others’ values, and balancing exchange. For instance, among these studies, Cheng et al. (2022) indicated that employees with greater degrees of positive reciprocity beliefs exhibit a stronger indirect influence of family-supportive boss conduct on unethical pro-family behavior via feelings of duty.

In contrast to earlier research, this study made the case that unfavorable reciprocity attitudes could make the linkage of abusive supervision with psychological distress worse. With this prior knowledge, the objective of this study is to close the knowledge gap regarding the linkage of abusive supervision with psychological distress via FNE, as well as how this relationship is influenced by negative reciprocity beliefs in the Egyptian hotel context.

## Literature review and hypotheses

### Theoretical underpinnings: Cognitive appraisal theory and social exchange theory

The impact of negative workplace stressors such as abusive supervision (Mawritz et al., 2014), supervisor undermining (Syed et al., 2018), workplace bullying (Majeed & Naseer, 2021), and exploitative leadership (Syed et al., 2021) on employee attitudinal and behavioral outcomes is mostly derived from cognitive appraisal theory (CAT) (Lazarus & Folkman, 1984). Cognitive appraisal refers to “*a process through which the person evaluates whether a particular encounter with the environment is relevant to his or her well-being, and if so, in what ways*” (Folkman et al., 1986, p. 992).

CAT theory emphasizes the sequence of the stressor-appraisal-emotion-outcome process. According to the theory, employees make a cognitive assessment of the factors that cause stress in the workplace. In this context, they first consider whether the factor causing stress will affect their well-being (called primary appraisal). In other words, they seek an answer to the question of whether the stressor is beneficial or harmful to them. Then, by entering a second appraisal process, they confirm in their mind whether the stressor is challenging or threatening. They decide how they will handle the stress at this stage. Previous research has shown that a challenging stressor has a whip role in an individual’s self-development. However, a hindrance stressor can be perceived as a threat to self-development (LePine et al., 2005).

In addition, previous studies indicate that the hindrance stressor positively affects negative emotions (e.g., anxiety and fear) (Lazarus & Folkman, 1984; Mawritz et al., 2014). In line with the theory, this study proposes that whenever employees experience abusive supervision, they may appraise the situation as threatening rather than challenging, which may result in a high level of psychological distress. This ultimately results in negative employee attitudinal and behavioral outcomes. For analyzing employee attitudes and actions in organizations, social exchange theory (SET) is the most cited theoretical frameworks (Cropanzano & Mitchell, 2005). According to this theory (Blau, 1964), the interaction between individuals within the organization is interdependent and is shaped by the words or actions of any party (including coworkers, supervisors, managers, etc.), which in turn determines the quality of the relationship among the parties (Cropanzano & Mitchell, 2005).

One of the key norms of the SET that guide the exchange processes is restricted reciprocity, which refers to one party’s action prompting another to respond. Individuals react similarly to the parties from which they gain or lose (Gouldner, 1960). Employees’ responses are mostly shaped by the treatment they receive from their supervisors. Abusive supervisors display hostile verbal or nonverbal behaviors towards the employees, including shouting at them, using aggressive eye contact, and withholding needed information, and others (Kim et al., 2015).

**Fig. 1:**
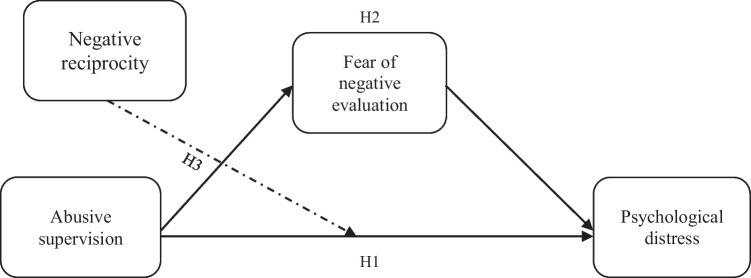
Conceptual model for moderated mediation

Employees may reciprocate the unfair treatment by their supervisors to nurture a balance of fairness, which may help to sustain the relationship (Koay et al., 2022). According to prior studies, people who hold strong negative reciprocity beliefs respond to abuse more strongly than those who hold weaker views (see Jahanzeb et al., 2019; Koay et al., 2022). We believe that SET could provide important insights into which individuals are more affected by abusive supervision in terms of psychological stress. We proposed that negative reciprocity beliefs may exacerbate the FNE’s role in the abusive supervision-employee psychological distress relationship. Figure 1 illustrates the conceptual model for moderated-mediation analysis of through FNE and negative reciprocity beliefs in the abusive supervision-psychological distress relationship.

### Abusive supervision and psychological distress

Tepper (2000) defined abusive supervision as “*subordinates’ perceptions of the extent to which supervisors engage in the sustained display of hostile verbal and nonverbal behaviors*” (p.178). Abusive supervision is distinguished by a subjective evaluation that employees learn through observing their superiors’ attitudes. The majority of its manifestations include compulsion, rudeness, outbursts of rage, and public condemnation (Bies, 2000). Previous studies have provided strong evidence that abusive supervision results in detrimental consequences to subordinates’ psychological health and their work-related behaviors in organizations (Bani-Melhem et al., 2021; Farooq & Sultana, 2021; Wang et al., 2022). The organization’s ability to survive and achieve long-term success depends critically on this crucial employee’s unfavorable reactions to abusive management. While abusive supervision is mostly detrimental to all organizations, service companies should attach great importance to it and find ways to overcome its negative outcomes. For instance, Lyu et al. (2016a) found that organizational identification mediated the negative linkage of abusive supervision with proactive service performance among hotel employees.

Park and Kim (2019) revealed that abusive supervision positively affected service sabotage in the hospitality context. Moreover, some characteristics of a leader may drive abusive supervision, including narcissism (Waldman et al., 2018), anger and anxiety (Mawritz et al., 2014), and an authoritarian leadership style (Kiazad et al., 2010). Organization-related antecedents include organizational norms such as aggressive norms and organizational sanctions against aggression (Zhang & Bednall, 2016). Subordinate-related antecedents include negative affectivity, supervisor-directed attribution, neuroticism, narcissism, and power distance (Tepper et al., 2006).

Finally, some key supervisor demographics (e.g., age, gender) and subordinate demographics (e.g., age, gender, tenure, working time with supervisor) drive supervisors to be abusive (Kim et al., 2022; Ouyang et al., 2015; Zhang & Bednall, 2016). According to Andrews and Slade’s (2001) first definition of psychological distress, it is a condition of emotional discomfort caused by obligations and pressures that are difficult to manage in daily life. In the context of employees and work relationships, Cadieux and Marchand (2014) defined psychological distress as identifying several workplace aspects related to the degree of psychological distress. These aspects include the use of talents, task diversity, social support at work, and benefits from the perception of being linked to reduced levels of psychological distress.

From the different available studies, different concepts have been associated with the prevalence of psychological distress. In particular, Tepper et al. (2007) noted that abusive supervision is the most common concept related to psychological distress. Other factors include long-term threats to employees’ well-being, including progressive unemployment, persistent financial worries, and relationship issues. Exposure to public criticism, coercion, and rudeness by supervisors is a stressful workplace action that deteriorates employees’ well-being (Xu et al., 2015). When employees are exposed to stressful actions that are derived from abusive supervision, they frequently have a negative state of mind, which is a sign of psychological distress (Park et al., 2018). Psychological distress emerges since individuals tend to return what they obtain from others in any social context (Chen et al., 2021).

Psychological distress has been linked to rudeness, hostility, humiliation, public criticism, wrath, and yelling, all of which lead to higher turnover, according to Raza et al. (2019). Moreover, there is a perceived decline in organizational justice. Tepper (2007) suggested that abusive supervision, which he defined as mocking, belittling, and shouting at subordinates, is the underlying cause of long-term psychological distress and has huge consequences for all employees, whether new or senior. The psychological distress that comes with coping with domestic violence issues is increased for the victims of abusive supervision. We develop the following hypothesis in light of these justifications:

- H1. There is a positive association between abusive supervision and psychological distress.

### Fear of negative evaluation

Fear is brought on by environmental factors that might be detrimental and serves as an internal early warning system that signals a need for action (Leary, 1983). Fear warns the individual of an impending threat and prepares the individual for action against this threat (Sweeney & Pine, 2004). However, when the individual considers that he/she cannot overcome this perceived threat, he/she experiences psychological and emotional distress (Satici et al., 2021; Siddiqi et al., 2022). Leary (1983) claimed that the concept of FNE encompasses feelings of anxiety about other assessments, pain over the poor evaluations, and anticipation that other people will also have unfavorable judgments about them. This is specifically related to the feeling of unfavorable evaluation that occurs when one is anticipating or engaging in socially inclined circumstances. These new challenges have been evaluated, and it is clear that abusive monitoring would be obvious and might cause employee distress.

A fear of criticism leads to the development and manifestation of anxieties that are more widespread anxiety disorders and psychopathologies (Carleton et al., 2006). This is identified as the apprehension and distress contributed by concerns about being judged disparagingly or hostilely by others. According to Syed et al. (2021), employees who work under an abusive supervisor may experience higher levels of social fear to be positively appraised. Moreover, subordinates who are exposed to abusive supervision cannot retaliate against their supervisor for fear of negative evaluation, which may ultimately lead to psychological distress (Shah et al., 2022). Rani et al. (2021) supported the linkage of abusive supervision with employee promotive voice behavior is mediated by paranoia arousal, which is a multidimensional concept and covers FNE. Since previous studies empirically supported the positive effect of FNE on psychological distress (Shafique et al., 2017) and supported evidence about the mediating role of FNE in the linkage of unethical leadership styles with employee outcomes. Hence, the current study proposes that FNE will mediate the abusive supervision-psychological distress linkage.

As indicated by Nonterah et al. (2015), improved psychological symptoms of anxiety and depression patients show that the value of negative evaluation has a mediating impact on the linkage of abusive supervision with psychological distress. According to Bhandarker and Rai (2019), aggressive coping, avoidance of coping, and adaptive coping all have a negative link with the loss of self-worth and ultimately lead to high-level employees’ suffering. Based on these justifications, we postulate that:

- H2. FNE mediates the positive effect of abusive supervision on psychological distress.

### Moderated-mediation effect

We suggest reciprocity as one of the core tenets of SCT theory (Blau, 1964), which may offer a thorough explanation of the focal role of FNE in the abusive supervision-psychological distress relationship. Reciprocity refers to "*a mutually gratifying pattern of exchanging goods and services*" (Gouldner, 1960, p. 170). While positive reciprocity is described as providing advantages to those who have previously provided such benefits to the other party, whereas negative reciprocity is described as taking revenge for the maltreatment the other party experienced (Cropanzano & Mitchell, 2005; Gouldner, 1960).

When employees are abused by their supervisors, they may seek justifiable revenge (Matejkowski et al., 2011). However, Gouldner (1960) argued that not every victim seeks revenge. Some who have been wronged may see that they deserve the mistreatment they see or may see that this mistreatment is a punishment for an injustice they have committed before. Therefore, employees may hold different opinions regarding whether negative reciprocity is appropriate (Mitchell & Ambrose, 2007). Previous research supported employee negative reciprocity’s moderating role in their research model. For instance, Ayub et al. (2021) found that employees with high levels of negative reciprocity are more likely to be affected by abusive supervision in terms of evasive concealing and acting dumb, two key aspects of knowledge hiding.

Wu et al. (2014) revealed that negative reciprocity moderated the workplace incivility-interpersonal deviance link. Koay et al. (2022) found that high levels of negative reciprocity, making abusive supervision have a stronger effect on cyberloafing. Jahanzeb et al. (2019) revealed that high levels of negative reciprocity make the negative linkage of abusive supervision with employee creativity via knowledge hiding strong. According to Yao et al. (2022), negative reciprocity moderates the linkage of workplace ostracism with employee silence, making the associations more prominent among employees who have significant negative reciprocity. Based on these theoretical explanations and empirical evidence, we argue that employees’ reciprocity beliefs moderate abusive supervision-psychological distress relationship. The severity of the abusive supervision-psychological distress relationship through FNE is also likely to be conditionally influenced by reciprocity beliefs, thereby indicating a pattern of moderated-mediation approach as depicted in Fig. 1.

- H3. Negative reciprocity moderates the indirect effect of abusive supervision on psychological distress via FNE.

## Methods

### Participants and pilot test

The concepts’ scales found in the theoretical model were translated from English into Arabic to fit the Egyptian dialect. To check content validity, eight experts were contacted to improve ambiguous sentences and make them easier for respondents to understand without violating the original content of the constructed items. Hence, their suggestions were taken into consideration. As such, a pre-test was performed using an online questionnaire, which was designed via the Google Form platform. This is due to the COVID-19 outbreak-related limitations enforced in Egypt, which prevented gatherings and imposed a curfew on most daily life hours.

A short link has been prepared that includes the purpose of the questionnaire and 40 items of the intended concepts, divided into four main sections. Besides, this questionnaire included the demographic characteristics of the respondents. To start the dispatch process, two parties were contacted to connect us with hotel employees: the Egyptian Hotels Association and MSc/Ph.D. students working in these hotels. They notified us with private emails and phone numbers via WhatsApp for the intended employees. A total of 52 out of 80 responses as a pilot test were received via custom author email that they completed this survey. These respondents confirmed their understanding of the items in every concept in the notes. This prompted us to move to the next step, which is defining the sampling, collecting the main data, and confirming whether the sample is sufficient to conduct statistical analysis.

Additionally, an open-ended method was used to provide participants with the chance to provide more information. Every participant was asked if they had ever seen an abusive supervisor target another employee. Employees have posed four similar open-ended questions. For example, “*How did you face abusive supervision behaviors in your workplace in terms of “braying up memories of your past failures and blunders, blaming you to shield him/her from shame?*” *and “How did your abusive behaviors affect your fear of others’ negative evaluation of you and your feelings of nervousness and hopelessness at this hotel?”.*

### Sampling and data collection

A non-probability convenience sampling approach was used in selecting respondents and establishing the sample sampling stages (Aaker et al., 1995). This sampling makes it simple to collect samples that are both affordable and efficient in terms of time and labor. Due to their high emotional demands and intense work schedules; hotel employees at five-star hotels were selected as the research population (Khliefat et al., 2021). In addition, their shift schedules and working hours are erratic and unpredictable (AlKayid et al., 2022). The main hotels are located in the five main tourist cities in Egypt: Hurghada, Greater Cairo, Sharm El-Sheikh, Luxor, and Aswan. These cities were chosen because they contain many resorts, hotels and tourist attractions in Egypt (Brown & Osman, 2017).

Since the vast majority of hotels had implemented stringent precautions to stop the spread of COVID-19 mutant pandemics, the online surveys were distributed in three waves. According to Booking.com, the majority of the hotels selected are 42 hotels. These establishments host the majority of summits, conferences, and international events for Arab and African countries, and because of the labor-intensive nature of their operations, they have been asked to voluntarily participate. However, 15 hotel management (representing 650 employees) verbally informed the consent. Consequently, 600 questionnaires were delivered (an average of 40 copies per hotel).

To lessen the likelihood of common method bias, the primary data were collected from mid-April to late July 2021 using the time-lag approach. Hence, the authors asked respondents about abusive supervision behaviors from April 25 to May 18 in Time1, and then about psychological distress related to these abusive behaviors from June 14–28 in Time2. Lastly, the authors asked respondents about the extent of their negative reciprocity and fear of others’ evaluation within Time3 from July 6–27, 2021. A set of MSc and Ph.D. students working in these hotels helped the custom author contact the same respondents throughout the data collection period. It was unnecessary to run any tests since even though a few values were missing, they accounted for less than 5% of the total.

Consequently, the p-value was negligible, indicating that the presence of missing data was merely coincidental (Tabachnick & Fidell, 2013). A total of 458 responses—representing a response rate of 76.33%—were received. The final sample included 412 valid cases after the data had been processed and responses that had significant outliers were omitted. The appropriateness of the sample size was evaluated using the Cohen (1992) rule-of-thumb, which states that a sample size of 412 substantially exceeds the minimum for 80% statistical power at a 5% level of significance.

Of the 412 respondents profiled, 61.8% of those polled were men, while 38.2% were women. Furthermore, 69.4% were unmarried, while 30.6% were married. In terms of age, 51.7% of respondents were between the ages of 20 and 29. The greatest educational levels attained by respondents were as following: 45.4% had a bachelor’s degree, while 32.5% had just finished high school. Furthermore, 24% of respondents had three to five years of professional experience, compared to 35.2% who had one to three years. The respondents work in the following departments: 28.9% in the kitchen; 19.4% in the restaurant; and 13.8% in both housekeeping and finance.

### Measurement instrument

All concepts were assessed using previously validated measures by combining multiple items from the existing literature (see Appendix Table 5).

#### Abusive supervision

Three items were adapted from Ampofo et al. (2022) to measure the degree to which employees felt abused by his/her immediate supervisor over the last month. One of its sample items stated: “My supervisor brined up memories of my past failures and blunders.” (α = 0.88).

#### Negative reciprocity

Four items were adopted using hotel employees’ self-report to rate their negative reciprocity in the workplace adapted from Matejkowski et al. (2011). A sample item includes “When my supervisor offends me, I will return the favor.” (α = 0.75).

#### Fear of negative evaluation

Ratings of employees’ fear of being negatively evaluating by others in the workplace, were obtained using eight items modified from Syed et al. (2021). Employees were asked to rate their agreement with statements like “It irritates me when others have a negative opinion about me” (α = 0.96).

#### Psychological distress

Anasori et al. (2021)’s scale was used to measure the degree to which employees felt psychological distress in their workplace during the past month. One of its sample items stated: “I am nervous.” (α = 0.93). A 7-point scale, with "*1* = *strongly disagree and 7* = *strongly agree*," was used by respondents to express their responses.

### Common method variance (CMV)

To prevent CMV in data collection, blocking the participant’s name so as not to conflict with his interrogation and the respondents’ information will never be disclosed except for research purposes (AlKayid et al., 2022), along with the fact that there were no definitive correct or incorrect responses to any items, was emphasized (Min et al., 2016). Following data collection, Harman’s single-factor test was performed. The findings indicated that the first factor explained 33.35% of the total variance. These results fall below the 50% cut-off threshold, as advised by Podsakoff et al. (2003). Hence, CMV was not a major issue in the current paper.

### Data normality

The data’s skewness and kurtosis were examined to determine the dataset’s normality (Tabachnick & Fidell, 2013). Nonetheless, Tabachnick and Fidell (2013) suggest that when the sample size is greater than 200, a little deviation from normalcy does not always result in a meaningful difference in the research findings. According to Akinwande et al. (2015), one method of determining multicollinearity is to use the variance inflation factor (VIF) test. A significant correlation that might be a major issue is indicated if a VIF value were between 5 and 10. As shown in Table 1, there was no multicollinearity among the independent variables because VIF values varied from 1.96 to 2.27. The skewness and kurtosis tests, as demonstrated by Orcan (2020), are one method for estimating multicollinearity. If the z-scores of skewness and kurtosis are less than 1.96, the data is deemed normal and devoid of multicollinearity. Therefore, the data were devoid of multicollinearity and tends to have a normal distribution (see Table 1).

**Table 1:** Matrix of correlation coefficients, normality and multicollinearity

Concepts		Mean	S.D	Sk	Ku	1	2	3	4	VIF
1	Abusive supervision	3.39	.92	.90	.38	1				
2	Fear of negative evaluation	3.09	1.35	.93	.52	.71^**^	1			1.96
3	Psychological distress	2.41	1.18	.83	.95	.73^**^	.67^**^	1		2.01
4	Reciprocity belief (Negative)	4.64	1.21	.66	.29	–.74^**^	–.65^**^	–.75^**^	1	2.27

*S.D* standard deviation, *Sk* skewness, *Ku* kurtosis, *VIF* variance inflation factor; ^**^p < .01

### Data endogeneity and homogeneity

Several approaches were used to guarantee that endogeneity “v*ariables not included in the model that may be connected to include variables*” was not a major problem in this work. Endogeneity can be caused by a variety of factors, including missing data, measurement error, and simultaneity (Greene, 2008). Besides, statistical approaches for detecting missing variables are unable to determine if additional types of omitted variables exist (Antonakis et al., 2014, p. 93). As a result, "*theory, theory, and more theory*" is the most crucial guidance (Antonakis & Dietz, 2011). The hypotheses were generated based on a thorough assessment of the literature, and all omitted variables were held constant. Furthermore, based on the detailed literature analysis and explanation of assumptions, reverse causality/simultaneity (i.e., an independent variable may be caused by the dependent variable) is not a problem in this study.

Finally, the homogeneity of variance assumptions, which indicate that population variances must be equal, must be satisfied. Levene’s test is used to check data homogeneity (George & Mallery, 2003). The result reveals that the significance value (p) of all demographic categories was greater than 0.05: p value_gender_ = 0.247, p value_marital status_ = 0.361, p value_age group_ = 0.566, p value_high education level_ = 0.189, p value_professional experience_ = 0.426, p value_departement_ = 0.653. This implies that the homogeneity of variance is satisfied (Tabachnick et al., 2007).

## Results

### Reliability and validity tests

As stated in Table 2, confirmatory factor analysis (CFA) in the AMOS v.25 software was used to examine the convergent validity of each construct. According to the CFA results, the default model did not suit the real data well (Brown, 2006). AGFI = 0.95 (> 0.9), *χ*^*2*^/df = 4.99 (1, < 5), TLI = 0.94 (> 0.9), NFI = 0.92 (> 0.9), RMR = 0.06 (< 0.080), RMSEA = 0.03 (< 0.05), CFI = 0.99 (> 0.9), and GFI = 0.95 (> 0.9), with loadings of five items [PDS3, PDS5, PDS6, PDS8, and PDS9] less than 0.70, omitting them. Hence, the CFA was performed again and the results were excellent: AGFI = 0.95 (> 0.9), *χ*^*2*^/df = 4.99 (1 < 5), TLI = 0.96 (> 0.9), NFI = 0.95 (> 0.9), RMR = 0.04 (< 0.080), RMSEA = 0.03 (< 0.05), CFI = 0.93 (> 0.9), and GFI = 0.96 (> 0.9), indicating process to the next step, reliability and validity testing.

**Table 2:** Confirmatory factor analysis results

Concepts	Items	Mean	Standard factor loadings	*t* value	Cronbach’s α	CR	AVE
Abusive supervision	ABS1	3.46	.74	–	.89	.91	.59
	ABS2	3.40	.71	13.73			
	ABS3	3.43	.75	14.49			
Fear of negative evaluation	FNE1	3.39	.70	–	.91	.92	.64
	FNE2	2.97	.71	22.73			
	FNE3	2.72	.75	22.17			
	FNE4	3.15	.76	22.42			
	FNE5	2.85	.73	20.83			
	FNE6	3.08	.73	21.10			
	FNE7	3.42	.81	21.26			
	FNE8	3.10	.73	17.56			
Psychological distress	PDS1	2.33	.83	–	.89	.90	.68
	PDS2	2.23	.80	22.55			
	*PDS3*	*1.32*	*.42*	*7.23*			
	PDS4	2.25	.81	22.34			
	*PDS5*	*1.89*	*.48*	*5.11*			
	*PDS6*	*1.56*	*.37*	*3.21*			
	PDS7	2.49	.80	18.10			
	*PDS8*	*1.32*	*.29*	*5.78*			
	*PDS9*	*1.46*	*.39*	*4.12*			
	PDS10	2.74	.79	15.34			
Reciprocity belief (Negative)	RBN1	4.47	.79	–	.88	.91	.63
	RBN2	4.86	.77	17.32			
	RBN3	4.67	.79	17.90			
	RBN4	4.34	.78	17.63			

Italicized items are omitted for being less than 0.7

Cronbach’s alpha values of each construct exceed 0.70, suggesting that this questionnaire has high internal consistency (Nunnally & Bernstein, 1994). Furthermore, the values of composite reliabilities (CRs) and the standard factor loadings (SFL) exceed 0.70, and the average variance extracted (AVEs) of each construct exceed 0.50, indicating satisfactory convergent validity (Hair et al., 2019). As shown in Table 1, all concepts had strong and substantial relationships. Furthermore, the lowest value of the square root of the AVE of each construct exceeds the highest value of the correlation coefficient between all constructs (see Table 1), supporting each construct’s discriminant validity.

### Hypotheses testing

Before testing the hypotheses, the overall model fit was checked using AMOS v.25.0 software. Hence, the overall model fit indices suggested that the data was acceptable and could be utilized to evaluate hypotheses (Brown, 2006): AGFI = 0.91 (> 0.90), *χ*^2^/df = 4.62 (1, < 5), TLI = 0.93 (> 0.9), NFI = 0.91 (> 0.9), RMR = 0.06 (< 0.08), RMSEA = 0.04 (< 0.05), CFI = 0.92 (> 0.9), and GFI = 0.91 (> 0.9), as shown in Table 1. Using the PROCESS 4.0 macro in SPSS (Models 4 and 5); the direct, mediating effect and moderated-meditation effects were tested. The bias-corrected bootstrap estimation method based on a 5000 resample was used to get the bias-corrected 95% confidence intervals (BCIs) for the direct and conditional effects (Hayes, 2022). When the 95% bootstrapped confidence interval does not include 0, all effects are significant.

**Fig. 2:**
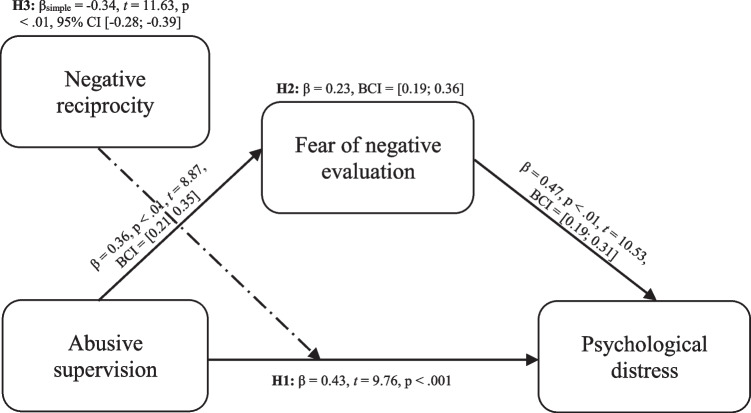
Structural equation modeling

Due to the following concerns, employing the bootstrapping approach proved effective. First, the effect size may be estimated without assuming that the variables or sample distribution have a normal distribution because it is non-parametric. Second, when working with low sample size, bootstrapping approach may be employed with more certainty than the non-bootstrapping approach (Hair et al., 2019). PROCESS macro v.4.0 using SPSS v.26.0 (MODEL 4) was employed (Hayes, 2022) to examine the mediator role of FNE in the abusive supervision-psychological distress association, as demonstrated in Table 3 and Fig. 2.

**Table 3:** Results of mediation effect (Model 4) and moderated mediation effect (Model 5)

	Mediation effect (Model 4)		Moderated mediation effect (Model 5)			
	*β*	SE	*t* value	*β*	SE	*t* value
*1. Mediator variable model (Fear of negative evaluation)*						
Constant	–0.67	0.021	–3.52^*^	–0.67	0.021	–3.52^*^
Abusive supervision	0.36	0.002	8.87^**^	0.36	0.002	8.87^**^
*R*^2^ = 31%				*R*^2^ = 31%		
*F* (7. 942) = 23.16^**^				*F* (7. 942) = 23.16^**^		
*2. Dependent variable model (Psychological distress)*						
Constant	3.15	0.18	20.21^*^	3.15	0.18	20.24^*^
Abusive supervision	0.43	0.004	9.76^**^	0.43	0.002	9.76^**^
Fear of negative evaluation	0.47	0.007	10.53^**^	0.47	0.008	10.44^**^
Negative reciprocity				-0.31	0.005	8.36^**^
Abusive supervision × Negative reciprocity				-0.25	0.021	9.11^*^
*R*^2^ = 42%				*R*^2^ = 46%		
*F* (6.561) = 28.66^**^				*F* (8.229) = 21.35^**^		
*PROCESS*			Effect	Boot SE	Boot LLCI	Boot ULCL
*3. Conditional indirect effect analysis at values of the moderator*						
Low			–0.18^**^	0.02	–0.13	–0.29
Mean			–0.26^**^	0.02	–0.18	–0.31
High			–0.34^**^	0.02	–0.28	–0.39

*ULCI* upper level confidence interval, *LLCI* lower level confidence interval;^**^p < 0.01, ^*^p < 0.05

The findings reveal that abusive supervision is positively associated with psychological distress (direct effect = 0.43, *t* = 9.76, p < 0.01) and FNE (β = 0.36, p < 0.01, *t* = 8.87, BCI = [0.21; 0.35]). FNE was positively related to psychological distress (β = 0.47, *t* = 10.53, p < 0.01, BCI = [0.19; 0.31]). According to the results of the 5000-time bootstrap approach, the indirect impact of psychological distress was significant (β = 0.23, 95% BCI = [0.19; 0.36]), and the total effect was 0.37, p < 0.01, 95% BCI = [0.36; 0.54]. Thereby, H1 and H2 were confirmed.

### Moderated-mediation model

**Fig. 3:**
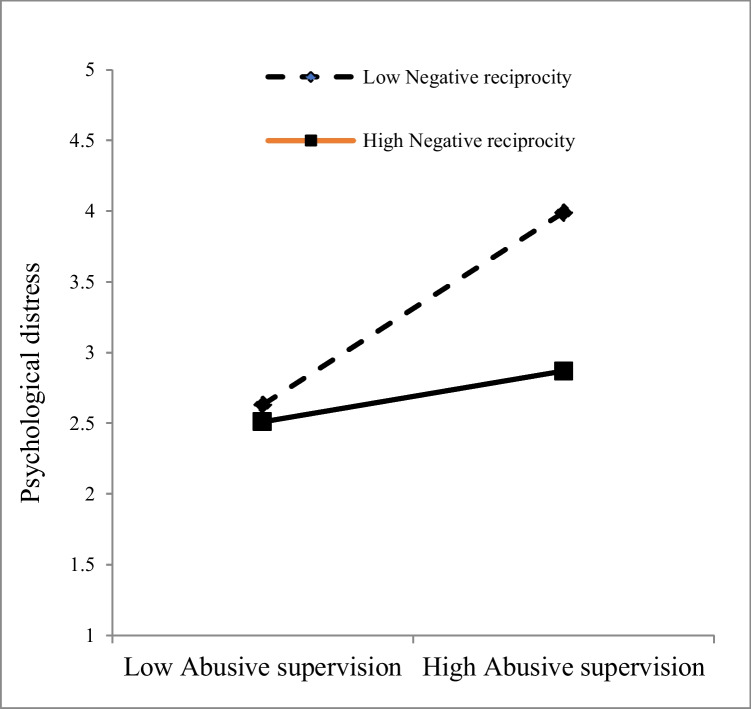
The moderating effect of negative reciprocity

Following Hayes (2022), the next step was to see if negative reciprocity could dampen the abusive supervision-psychological distress association through FNE (PROCESS macro v.4.0) in SPSS with Model 5. Table 3 displays the findings of the moderated mediation analysis. According to Table 3, the strong positive link among abusive supervision and psychological distress remained significant (β = 0.43, p < 0.01). As a result, the interaction of abusive supervision with negative reciprocity was plotted at various levels (see Fig. 3).

Significant interactions were discovered among abusive supervision and negative reciprocity (β = -0.25, *t* = 9.11, p < 0.05, BCI = [-0.12; -0.26], indicating that negative reciprocity dampened the linkage of abusive supervision with psychological distress. Hence, the positive linkage of abusive supervision with psychological distress are weaker in employees who hold a high level of negative reciprocity, but the intensity is stronger (-1SD of the mean; β_simple_ = -0.34, *t* = 11.63, p < 0.01, CI = -0.28; -0.39). The positive linkage of abusive supervision with psychological distress is stronger with a low level of negative reciprocity, although the intensity is weaker (+ 1SD of the mean; β_simple_ = -0.18, *t* = 7.21, p < 0.01, CI = 0.13; 0.29). As a result, H3 is supported. The results in Fig. 3 and Table 3 reveal that reciprocity belief has an antagonistic moderating effect because it reverses the positive effect of abusive supervision as a predictor of psychological distress through FNE.

### Narratology approach

The results of open-ended questions were interpreted using the narratology approach to determine which employees face these abusive behaviors and their reactions. Codes were assigned to the 412 replies to the open-ended questions, and the authors discovered that 84% of them discussed the following question: “*Brined up memories of my past failures and blunders*.” The following responses to the questions: “*Did not credit me for jobs that required a lot of effort*” and “*Blamed me to shield him/her from shame*” were mentioned by small percentages of respondents (see Table 4). Of the 412 respondents who provided open-ended responses, 52% discussed the impacts of abusive supervision on their fear of others’ negative evaluations; 27% went into detail about the effects of abusive supervision on their nervousness and hopelessness and 21% discussed the effects of abusive supervision in detail on feelings of nervousness and hopelessness in the presence of their FNE and negative reciprocity (see Table 4).

**Table 4:** Samples of qualitative responses to open-ended questions

How you faced abusive behaviors by your supervisor?		
Brined up memories of my past failures and blunders	Did not credit me for jobs that require a lot of effort	Blamed me to shield himself/herself from shame
I attempt to reply logically and with wisdom to my supervisor’s issue. I refrain from yelling or threatening her. Moreover, I avoid receiving too much attention from other people, exhibit emotional restraint, establish my worth and existence via my accomplishments, and do not let other people make me lose confidence	I do not express my annoyance and irritation with my supervisor’s mannerisms. I take note of what my supervisor appreciates in others and imitate it to maintain my productivity at work; along with I make an effort to think positively and creatively about how to enhance interpersonal interactions	I maintain calm when speaking with my supervisors to avoid arguing, getting annoyed, or irate, and I make sure my response is succinct, straightforward, and clear because my supervisors would not keep quiet about the lengthy conversations or insinuations
Abusive supervision behaviors affecting my ………………….		
Fear of others’ negative evaluation	Feelings of nervousness and hopelessness	Negative reciprocity in the presence of fear of others’ negative evaluation and feelings of nervousness and hopelessness
I worry about what others may think of me. Because of how they feel about me, I get irate with other people. I worry that people will not like me. In addition, I detest speaking in front of others because I fear their judgment. When people criticize me, I feel awkward. I worry about making a mistake. I also give too much thought to what others may think of me	I experience psychological instability due to a lack of self-awareness, extreme fatigue while working, and a sense of dissatisfaction with my position. I also care about having unfavorable expectations for life at work. These feelings make me feel hopeless and depressed	Due to my supervisor’s mistreatment, I feel offended, which causes me to experience tension, anxiety, and dread over other people’s opinions of me as well as worry over an offending response to offensive conduct. If my boss treats me like my coworkers, I feel content with life, psychological stability, and a sense of psychological satisfaction. If, however, I am sad, anxious, and unsatisfied with the execution of my obligations at work, I will complete the things assigned to me promptly

## Discussion

### General discussion

Employees are considered the most valuable assets that firms have because of the following characteristics: knowledge, experience, capabilities, ideas, visions, and views (Dirican & Erdil, 2020). Employees, on the other hand, may be exposed to a range of variables at work that can either positively or negatively impact their behavior, resulting in abusive supervision (Khan et al., 2016). As a result, academics have recently focused their attention on this phenomenon, investigating its sources and ramifications, as well as the intermediary processes and influencing elements. As a result, abusive supervision happens when supervisors repeatedly engage in hostile behavior against their employees (Jain et al., 2021). According to the findings, negative reciprocity had a moderating role in the abusive supervision-FNE linkage. As a result, this might be explained by the fact that reciprocity creates positive awareness among employees, reducing their FNE under abusive supervision.

Psychological distress is one of the most common symptoms among employees, which can be induced by a range of factors, including abusive supervision (Cadieux & Marchand, 2014). We examined linking abusive supervision with psychological distress among hotel employees. The findings also revealed that abusive supervision improved the psychological discomfort of hotel employees (Park et al., 2018). This finding supports CAT theory (Lazarus & Folkman, 1984). As a workplace stressor, employees may consider that abusive acts by supervisors have the potential to deteriorate their well-being. When they consider that they cannot struggle with the stressor (abusive supervision) due to the positional power of supervisors, their situation may result in some negative emotions such as fear, which ultimately leads to psychological distress. However, it was revealed that employees’ negative reciprocity moderates the abusive supervision-psychological distress link. Employees’ psychological distress was reduced in the presence of negative reciprocity, even though the strong correlation between abusive supervision and psychological distress appeared to be decreasing as the degree of negative reciprocity increased.

Our findings showed that the reciprocity attitudes of hotel employees had a negative effect on their mental health. It is possible to describe SET theory, a social psychology theory that states employee behavior and social stability as the result of multi-party bargaining (Iqbal & Rasheed, 2019). The findings related to the moderating role of negative reciprocity supports SET theory (Blau, 1964; Cropanzano & Mitchell, 2005). Employees may respond in the same manner to the treatment they received from their supervisor. Since abusive supervisors show hostile verbal or nonverbal behaviors to employees, employees may consider that they have the right to reciprocate the mistreatment.

According to the findings, employees’ fear of receiving a negative rating from others causes psychological distress. As believed by Nonterah et al. (2015), FNE is positively related to the theory influence on mental problems, including depression, anxiety, and stress. Under the umbrella of CAT theory, negative reciprocity beliefs were examined in the current paper to see if they had any effect on the association between psychological distress and FNE. Hotel employees with higher negative reciprocity were less likely to experience psychological distress as a result of their FNE.

### Theoretical implications

This study advances both academic and practical knowledge in the hospitality sector. Theoretically, the topic of this research has lately gained ground and is posing challenges for the study of psychology and social studies. It is so challenging to teach about applying hard skills to counter abusive supervision because of its strong tie to affecting business management and effectiveness. Still, one of the research addressing contemporary ideas (such as abusive supervision, FNE, and psychological distress) in the organizational behavior and mental health literature relevant to the hotel setting gives this paper its originality by integrating CAT and SCT theories. Given this, it is challenging to argue that these notions don’t exist in the Egyptian and Arab contexts by attempting to understand how they are related. Researchers should investigate any linkages between abusive supervision and the analysis of the behavioral reactions of hotel employees, according to the claims made by Raza et al. (2019).

In addition, the paper’s handling of negative reciprocity as a moderation-mediation for a deeper and more accurate explanation of this phenomenon and establishing the linking abusive supervision with psychological distress via FNE contribute to this paper’s significance. Specifically, it has been established by a lot of earlier research that testing meditation in the context of abusive supervision, psychological disorders, and anxiety over poor evaluation is rare and yet in its adolescence (Li et al., 2017; Mitchell & Ambrose, 2007). Additionally, there is a glaring dearth of understanding regarding the mediating processes that explain these linkages as well as the phenomena of abusive supervision as compared to the other employed conceptions at the level of Arab studies. This study, which is a new addition to organizational behavior theories, fills certain information gaps concerning the examination of links between abusive supervision and psychological discomfort among hotel employees by highlighting an essential occurrence that occurs in the Arab setting.

Individuals often react predictably to workplace events, according to SET theory. Because people often reciprocate what they receive from others in whatever social situation, including the workplace, distress develops (Iqbal & Rasheed, 2019). According to CAT theory, being subjected to abusive supervision at the same time as feeling frightened, scared, or anxious about displaying an aggressive or violent setting impairs social behaviors. These consist of criticizing, demoralizing, and ignoring. Employees who are subjected to abusive management, on the other hand, frequently retaliate by acting out. The findings of this paper support the two theories that are combined to serve the research objectives and to fill a research gap left by earlier studies, which represents a theoretical contribution to the current research.

### Managerial implications

The results benefit a large aspect of society, particularly Egypt’s hotel sector. First, decision-makers in the hotel industry are anticipated to receive useful information from this article regarding the essential role that employees’ beliefs about negative reciprocity play in helping to alleviate psychological distress and FNE. Second, our findings provide a compelling argument to Egyptian hotel administrations for the need to continuously evaluate the effectiveness of supervisors, with a particular emphasis on appraisal aspects related to the psychological aspects of transactions, such as how subordinates are treated. For instance, a model created to assess the performance of supervisors is emotionally oriented to gauge the leader-subordinate relationship (Akhtar et al., 2021).

Third, since the majority of organizations are focused on material performance, it is well recognized that moral factors, such as psychological distress and FNE due to abusive supervision, are challenging to quantify. The current research makes a significant scientific contribution to the hotel industry by exposing these factors that could influence employees’ performance. Furthermore, it is challenging to quantify independent factors without conducting in-depth interviews with employees to uncover those issues (Shum, 2021).

Fourth, creating training programs that enhance supervisors’ effectiveness in their interactions with subordinates is crucial, according to the researchers’ interpretation of the research’s new findings. The findings indicated that service failures, an inability to concentrate, being late for work, and conflicts with coworkers are all negatively affected by abusive supervision and psychological pressures, including sadness, anxiety, and tension. Because service organizations depend on satisfied and pleased personnel, this will eventually have a negative effect on hotel performance (Khalid et al., 2020; Raza et al., 2019).

Finally, it should be mentioned that this study provides a strong indicator for hotel management to focus on and support quick resolution of the psychological and behavioral aspects of superior-subordinate interactions. The development of educational programs for managers and supervisors about the consequences of abusive supervision on their subordinates’ mental health may result in them.

### Limitations and further studies

This study has certain limitations, even though it makes some helpful theoretical and practical contributions. First, Research data from Egyptian five-star hotel employees were provided, which may limit how far the findings could be generalized. As a result, future researchers might employ a multi-tiered strategy when examining the connection between the superior and the subordinate from the perspective of hotel leaders. Future research might be carried out in restaurants, travel agencies, and resorts in other places, whether in Africa or abroad. Second, without respect to management level or other factors, the focus was on employees working in various departments of five-star hotels. Future research should consider other administrative levels, such as the chief executive and middle leaders. Third, this study ignored interviews in favor of a quantitative, questionnaire-based method. Therefore, the focus-groups approach should be used in the future.

Fourth, the authors gathered field data during the height of the COVID-19 pandemic, which necessitated that they do so at different times since hotel management implemented rules to restrict daily labor volume to handle the issue. Data collection should be the main focus of future studies post-COVID-19. This study relies on prior theoretical studies’ relationships between a variety of factors, including abusive supervision, FNE, and psychological distress. Future studies might investigate the mediating role of health education in domains such as psychological distress alleviation, prioritizing approaches, and time management. Finally, the authors did not examine the measurement model’s control variables. Furthermore, the majority of responses were from young people under the age of 30. As a result, future models can include gender, age, and educational level to do multi-group analysis between their categories.

### Electronic supplementary material

Below is the link to the electronic supplementary material.

Supplementary file1 (DOCX 47 KB)

## Data Availability

The datasets analyzed during the current study are available from the corresponding author upon reasonable request.

## Appendix

**Table 5:** Measurement items

Concepts	Items	Description
Abusive supervision	ABS1	My supervisor brined up memories of my past failures and blunders
	ABS2	My supervisor did not credit me for jobs that require a lot of effort
	ABS3	My supervisor blamed me to shield himself/herself from shame
Fear of negative evaluation	FNE1	I am concerned about what others think of me, even though I know it does not matter
	FNE2	It irritates me when others have a negative opinion about me
	FNE3	I am frequently concerned about others recognizing my flaws
	FNE4	I am concerned about the impression I am leaving on others
	FNE5	I am scared that others will not like me
	FNE6	I am worried others will discover something wrong with me
	FNE7	I am worried about what others think of me
	FNE8	I am constantly concerned that I will say or do the wrong thing
Psychological distress	PDS1	I felt faintness or weakness
	PDS2	I felt tense or keyed up. [Omitted]
	PDS3	I blamed myself for things
	PDS4	Everything was an effort for me. [Omitted]
	PDS5	I felt blue. [Omitted]
	PDS6	I felt frightened
	PDS7	I felt worthlessness. [Omitted]
	PDS8	I felt everything was an effort. [Omitted]
	PDS9	I felt hopeless about the future
	PDS10	I felt dizziness
Reciprocity belief (Negative)	RBN1	If my supervisor acts nicely around me, I am polite and nice; if not, it’s tit-for-tat
	RBN2	When my supervisor offends me, I will return the favor
	RBN3	When my supervisor is rude to me, I also start acting rudely
	RBN4	The way my supervisor treats me greatly affects how I treat them
