# Supplementary material for: A moderated-mediation analysis of abusive supervision, fear of negative evaluation and psychological distress among Egyptian hotel employees
Source: Curr Psychol. 2022 Oct 26;42(4):3395–410. doi: 10.1007/s12144-022-03822-4 (PMC9607795; doi:10.1007/s12144-022-03822-4)
Supplement: Supplementary file 1 — Supplementary file1 (DOCX 47 KB) [file 12144_2022_3822_MOESM1_ESM.docx]

**Supplement A.** Survey questionnaire.

**Dear Employee,**

We would like to kindly request your participation in this survey, which will take less than 15 minutes to complete. Note that your honest feedback is highly welcomed. Finally, your information will remain confidential and will only be used for scientific research purposes.

| **Section 1. Beliefs in Reciprocity**  (Please Shade the appropriate box) | | | | | | | |
| --- | --- | --- | --- | --- | --- | --- | --- |
| Please rate the extent of your belief in reciprocity in the workplace | Strongly agree | Agree | Somewhat agree | Moderated | Somewhat disagree | Disagree | Strongly disagree |
| Helping someone is the best way to ensure that she or he will help me in the future. | □ | □ | □ | □ | □ | □ | □ |
| I do not misbehave with others to avoid their misbehaving with me. | □ | □ | □ | □ | □ | □ | □ |
| I am afraid of the reactions of someone I have previously mistreated. | □ | □ | □ | □ | □ | □ | □ |
| I believe that if I work hard, I will be rewarded. | □ | □ | □ | □ | □ | □ | □ |

| **Section 2. Psychological Distress**  (Please Shade the appropriate box) | | | | | | | |
| --- | --- | --- | --- | --- | --- | --- | --- |
| Please indicate the degree which you felt psychological distress in your workplace during the past month | Always | Very often | Often | Sometimes | Rarely | Almost never | Never |
| I felt faintness or weakness. | □ | □ | □ | □ | □ | □ | □ |
| I felt tense or keyed up. | □ | □ | □ | □ | □ | □ | □ |
| I blamed myself for things. | □ | □ | □ | □ | □ | □ | □ |
| Everything was an effort for me. | □ | □ | □ | □ | □ | □ | □ |
| I felt blue. | □ | □ | □ | □ | □ | □ | □ |
| I felt frightened. | □ | □ | □ | □ | □ | □ | □ |
| I felt worthlessness. | □ | □ | □ | □ | □ | □ | □ |
| I felt everything was an effort. | □ | □ | □ | □ | □ | □ | □ |
| I felt hopeless about the future. | □ | □ | □ | □ | □ | □ | □ |
| I felt dizziness. | □ | □ | □ | □ | □ | □ | □ |

| **Section 4. Abusive Supervision**  (Please Shade the appropriate box) | | | | | | | |
| --- | --- | --- | --- | --- | --- | --- | --- |
| Please indicate the degree which you feel abuse by your immediate supervisor | Strongly agree | Agree | Somewhat agree | Moderated | Somewhat disagree | Disagree | Strongly disagree |
| My supervisor brined up memories of my past failures and blunders. | □ | □ | □ | □ | □ | □ | □ |
| My supervisor did not credit me for jobs that require a lot of effort. | □ | □ | □ | □ | □ | □ | □ |
| My supervisor blamed me to shield himself/herself from shame. | □ | □ | □ | □ | □ | □ | □ |

| **Section 3. Fear of Negative Evaluation**  (Please Shade the appropriate box) | | | | | | | |
| --- | --- | --- | --- | --- | --- | --- | --- |
| Please rate your fear of being negatively evaluated by others for you in the workplace | Strongly agree | Agree | Somewhat agree | Moderated | Somewhat disagree | Disagree | Strongly disagree |
| I am concerned about what others think of me, even though I know it does not matter. | □ | □ | □ | □ | □ | □ | □ |
| It irritates me when others have a negative opinion about me. | □ | □ | □ | □ | □ | □ | □ |
| I am frequently concerned about others recognizing my flaws. | □ | □ | □ | □ | □ | □ | □ |
| I am concerned about the impression I am leaving on others. | □ | □ | □ | □ | □ | □ | □ |
| I am scared that others will not like me. | □ | □ | □ | □ | □ | □ | □ |
| I am worried others will discover something wrong with me. | □ | □ | □ | □ | □ | □ | □ |
| I am worried about what others think of me. | □ | □ | □ | □ | □ | □ | □ |
| I am constantly concerned that I will say or do the wrong thing. | □ | □ | □ | □ | □ | □ | □ |

| **Respondents Profile**  (Shade the appropriate choose for you) |
| --- |
| **Gender**  □ Male □ Female |
| **Marital status**  □ Single □ Married |
| **Age**  □ 20-29 □ 30-39 □ 40-49 □ 50 above |
| **High education level**  □ M.Sc./Ph.D. □ Diploma □ Bachelor □ High school □ Preparatory school □ Other |
| **Professional experience**  □ < 1 year □ 1 to < 3 year □ 3 to < 5 years □ ≥ 5 years |
| **Department**  □ Reception □ Kitchen □ Restaurant □ Room service □ Housekeeping □ Finance □ Other |

**Open-ended questions**

1. How you faced abusive supervision behaviors in your workplace? Describe it in terms of mocked your efforts, intruded on your family privacy, prevented your interacting with others, and diminished you in the others’ eyes.

2. How did your abusive behaviors affect your beliefs in reciprocity at this hotel?

3. How did your abusive behaviors affect your fear of others’ negative evaluation of you at this hotel?

4. How did your abusive behaviors affect your feelings of nervousness and hopelessness at this hotel?

Thank you for participating in the questionnaire. Please feel free to write any comments down.

**Supplement B.** Respondents profile.

| % | Frequency (N= 412) | Category |
| --- | --- | --- |
|  |  |  |
|  |  | Gender |
| 61.8 | 254 | Male |
| 38.2 | 158 | Female |
|  |  | Marital status |
| 69.4 | 286 | Single |
| 30.6 | 126 | Married |
|  |  | Age group (years) |
| 51.7 | 213 | 20-29 |
| 26.2 | 108 | 30-39 |
| 13.8 | 57 | 40-49 |
| 8.3 | 34 | 50 above |
| High education level | | |
| 3.6 | 15 | M.Sc. / Ph.D. degree |
| 15.3 | 63 | Diploma |
| 45.4 | 187 | Bachelor degree |
| 32.5 | 134 | High school |
| 2.2 | 9 | Preparatory school |
| 1.0 | 4 | Other |
| Professional experience | | |
| 29.1 | 120 | < 1 year |
| 35.2 | 145 | 1 to < 3 years |
| 24.0 | 99 | 3 to < 5 years |
| 11.7 | 48 | ≥ 5 years |
|  |  | Department |
| 8.3 | 34 | Reception |
| 28.9 | 119 | Kitchen |
| 19.4 | 80 | Restaurant |
| 12.6 | 52 | Room service |
| 13.8 | 57 | Housekeeping |
| 13.8 | 57 | Finance |
| 3.2 | 13 | Other |
